# Cyclic Catamenial Dermatoses

**DOI:** 10.1155/2013/156459

**Published:** 2013-10-02

**Authors:** Trinh Hermanns-Lê, Jean-François Hermanns, Marianne Lesuisse, Gérald E. Piérard

**Affiliations:** ^1^Department of Dermatopathology, Unilab Lg, University Hospital of Liège, 4000 Liège, Belgium; ^2^Department of Dermatology, Diagnostic Centre, 4800 Verviers, Belgium; ^3^Department of Dermatology, Regional Hospital Citadelle, 4000 Liège, Belgium; ^4^Laboratory of Skin Bioengineering and Imaging, Department of Clinical Sciences, B23, University of Liège, 4000 Liège, Belgium

## Abstract

Circulating sex hormones follow major fluctuations during the ovarian cycle. The so-called premenstrual syndrome represents a global condition grouping the diversity of catamenial disorders. At the skin level, the sebaceous gland activity is obviously modulated by these endocrine fluctuations. In addition, a series of pathological manifestations take place simultaneously in some women. Among them, the most frequent skin condition is represented by catamenial acne. Concurrently, the autoimmune progesterone dermatitis refers to a diversity of skin alterations resulting from an immune reaction to progesterone. It is present under variable clinical aspects. A series of other recurrent skin conditions are not specifically induced but are merely exacerbated at the end of the ovarian cycle.

## 1. Introduction

Cyclic premenstrual physiological changes in healthy skin and various dermatoses represent common ailments. They most likely reflect the direct or indirect skin responses to fluctuations in circulating sex steroid hormones. Clearly, a series of chronic dermatoses show a transient worsening during the premenstrual phase of the ovarian cycle, while certain catamenial eruptions are specifically restricted to only the menstruation periods. The term autoimmune progesterone dermatitis (AIPD) refers to the rare skin disorders typically supported by progesterone hypersensitivity [1, 2]. These cyclic premenstrual dermatoses are cleared at menopause.

 This review aims at raising awareness about problems raised by a few skin disorders influenced by the catamenial phase of the ovarian cycle.

## 2. Sex Hormones, the Ovarian Cycle and the Skin

In diverse organs, sex hormones control the ovarian cycle and some related functions. Of note, skin contains receptors to estrogens, progesterone, and androgens. This organ is highly sensitive to the effects of these sex steroids. In particular, a series of premenstrual deteriorations of dermatoses are expected to represent some effects of progesterone representing the predominant circulating hormone at that time of the periodic ovarian cycle. 

**Figure 1:**
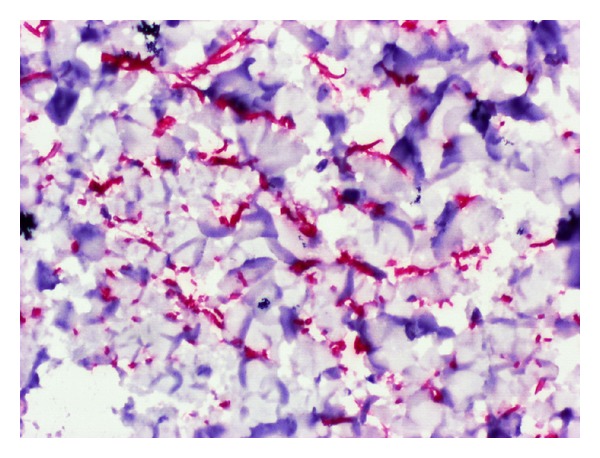
Dermal cells enriched in versican (immunohistochemistry anti-versican antibody).

 Estrogens somewhat mitigate the sebaceous gland activity. They possibly increase the density in intracellular dermal versican (Figure 1) and in extracellular hyaluronic acid. This pattern results in an increased hydration eventually leading to tissue water retention and turgescence. In rare instances, estrogens stimulate the intraepidermal melanogenesis, accounting for a possible transient patchy hyperpigmentation around the eyelids and nipples during the premenstrual phase. Progesterone effects on the skin are less firmly established. During the mid part of the ovarian cycle, the sebaceous gland activity is boosted, producing seborrhea and possibly mild acne. Skin vascularity is apparently increased during the second part of the ovarian cycle.

## 3. Premenstrual Syndrome

Diverse signs and symptoms including skin changes commonly develop during the premenstrual phase. They are collectively gathered under the title of premenstrual syndrome (Table 1). A specific and unique molecular background for the premenstrual syndrome has not yet been identified. It remains that fluctuations in endorphins, prostaglandins, prolactin, and progesterone have been evoked. Several hypotheses suggesting a progesterone-related effect were launched, although not yet validated. These include progesterone deficiency, a relative imbalance of estrogen and progesterone levels or a progesterone immune reaction. Although progesterone is commonly administered to control some aspects of the premenstrual syndrome associated with a functional deficit in natural progesterone, it has currently no place in the management of most catamenial skin conditions.

### 3.1. Catamenial Sebum Production

**Figure 2:**
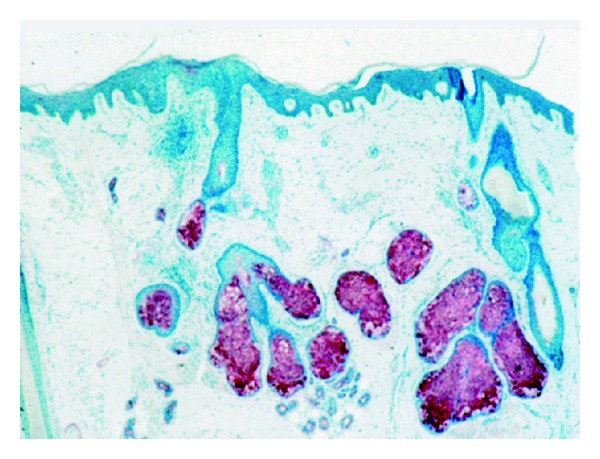
Sebaceous gland lobules (immunohistochemistry, anti-epithelial membrane antigen antibody).

Sebaceous glands are privileged targets for sex steroids, particularly 5*α*-dihydroxytestosterone (Figure 2). In addition, other hormones and neuropeptides are active on sebocytes. Sebum excretion on facial skin and scalp varies widely among individuals. In women, objective and controlled studies assessed the amount of sebum poured out at the skin surface. In addition, in many instances, the limited number of subjects precluded any sound conclusion. Estrogens at high dosages unquestionably reduce human sebaceous secretion. It remains debatable, however, whether they have any profound effect at the physiological levels and whether they play any sizeable part in normal control of the gland. Indeed, hormone contraceptives only exhibit a moderate sebosuppressive activity in acne-prone young women suffering from moderately increased seborrhea. It is possible that hormone replacement therapy (HRT) exerts no effect when seborrhea is absent or discrete. This does not preclude any possible effect in severe cases. Anyway, chronological aging by itself likely mitigates seborrhea.

### 3.2. Climacteric Sebum Production

Some studies showed that sebum excretion decreases with aging. Sebaceous glands are androgen targets exhibiting a large androgen receptor density in human skin. During climacteric aging, possible changes are expected in sebocyte proliferation, intracellular lipid synthesis, and sebum transit time in the infundibulum storage reservoir, as well as in sebum rheology and capture at the skin surface and inside the stratum corneum [3]. In particular, the sebum output at the skin surface in menopausal women appeared to be lower than that in younger nonmenopausal women [4]. By contrast, it was claimed to be increased in menopausal women under HRT [4, 5]. Contrasting findings were reported in another controlled study involving large numbers of women [3]. 

Modifications in the balance of sex hormones at menopause probably initiate changes in sebum physiology. The major decline in estrogen combined with a minimal decrease in androgens leads to a relative increase in the androgen-estrogen ratio. Such hormonal changes potentially affect several segments of the sebaceous follicle, in particular the volume of the sebum reservoir. This is associated with the progressive enlargement of the follicular openings [3]. Although sex steroids are tentatively offered as agents responsible for the objective changes, other hormonal and nonhormonal aspects of aging cannot be dismissed.

Any sebum excretion changes in postmenopausal women are more likely to be related to hormones than to age [3, 6]. In clinical trials, a large diversity prevailed among individual values of sebum output at the skin surface. In menopausal women out of HRT, a significant decline in sebum excretion rate accompanied by an increase in both the sebum replacement time and the mean sebaceous pore size was evidenced during the first postmenopausal decade [3]. The sebum excretion rate and casual level showed a wide range in interindividual differences soon after menopause. These physiological changes were less dramatic in women under HRT. It was concluded that postmenopausal aging affected sebum production, but HRT did not significantly control the complex process of seborrhea. However, HRT-recipient women showed less prominent variations in sebum output [3]. Clearly, the benefit in sebum regulation differed among women and remained hardly predictable. In some instances, HRT increased the casual sebum level [3], but there is a lack of consensus about that aspect. HRT was reported to mitigate the progressive enlargement of the openings of the sebum follicular reservoir [3]. The follicular pores remained narrow compared to the skin of nonsupplemented women. In these menopausal women, sebum excretion generally increased during the perimenopause and later on declined with chronologic aging [4]. 

The effect of climacteric and postmenopause upon the sebaceous gland function has not been thoroughly and adequately assessed using precise biometrological methods. The sebum dynamics varies throughout the adult life. In women, the average sebum production apparently remains almost stable over about three decades and drops significantly in the 50-odd years of age. However, this concept has been challenged.

### 3.3. Catamenial Acne

**Figure 3:**
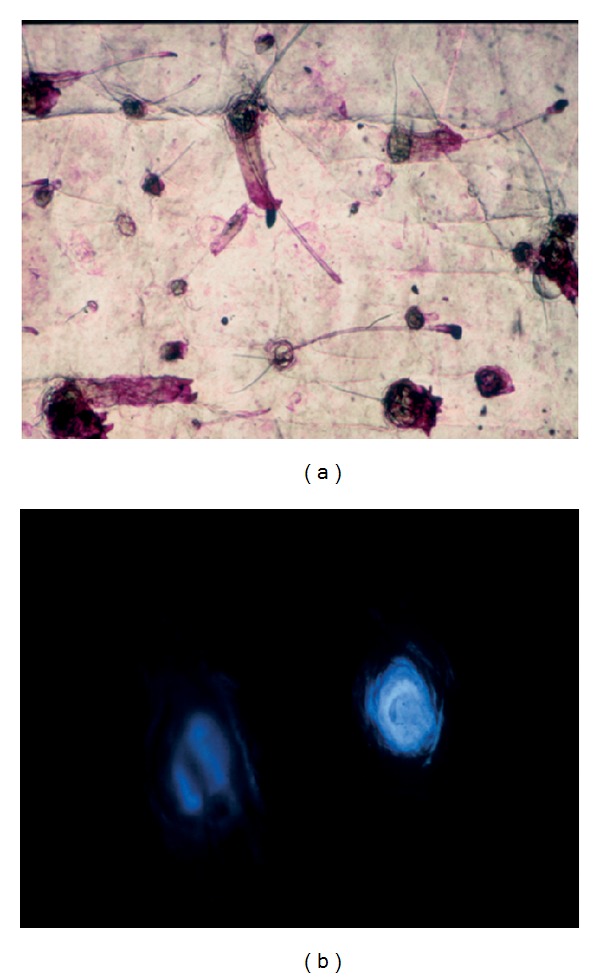
Cyanoacrylate skin surface strippings in catamenial acne. (a) Regular observation by optical microscopy revealing microcomedones. (b) Observation under fluorescence microscopy revealing fluorescent acro-infundibulum probably due to porphyrin released by *Propionibacterium acnes*.

**Figure 4:**
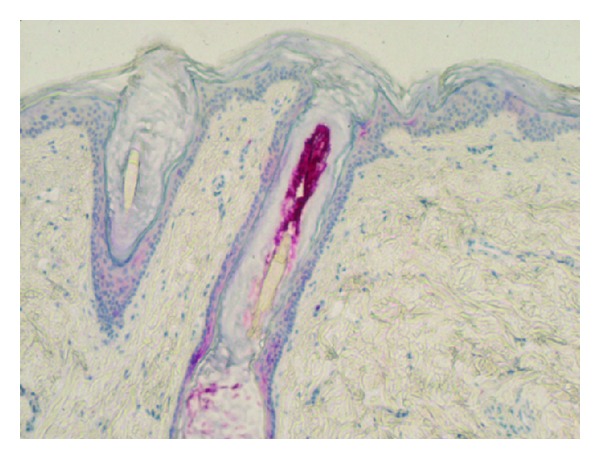
Microorganisms present in a follicular infundibulum in acne.

Catamenial acne consists of a crop of follicular papulopustules supervening in successive perimenstrual periods. By contrast, as shown by cyanoacrylate skin surface strippings, microcomedones and *Propionibacterium acnes* are present in a stable pattern unmodified by the ovarian cycle. This minimally invasive method reveals the heterogeneous distribution pattern of microcomedones on facial skin (Figure 3(a)). Such an aspect is unmodified during each ovarian cycle. If an immediate effect is not disclosed by such method, it remains that the cyclic repetition of any disturbance in the hormonal impact on the sebaceous hair follicle sustains a progressive worsening on the microcomedo generation. Similarly, the follicular fluorescence induced by porphyrin production by *P. acnes* is unmodified by the catamenial hormonal fluctuations (Figure 3(b)). Indeed, the microorganisms involved in the acne process are confined inside the sebaceous hair follicle infundibulum (Figure 4) and are not directly under hormonal influence.

Mild facial catamenial acne affects a number of women during the premenstrual period and is often accompanied by increased seborrhea of the scalp [7, 8]. A series of topical agents are usually effective for controlling catamenial acne [9]. In addition, suppression of both ovulation and postovulatory rises in progesterone levels is effective. Hence, some oral contraceptives are recommended [10, 11]. They help raising the levels of sex-hormone-binding globulin and thus reduce free testosterone and provide a clinical antiandrogenic effect. Some synthetic progesterone derivatives tend to worsen acne and should be avoided in acne-prone women. Physical treatments using light/laser sources appear as convenient modalities applicable to catamenial acne [12].

### 3.4. Catamenial Exacerbation of Preexisting Dermatoses

Cyclic premenstrual worsening of specific preexisting dermatoses is common in some women (Table 2). Both increased cutaneous vascularity and dermal edema, as well as the increased metabolic premenstrual activity, aggravate most pruritic conditions such as eczema and pruritus vulvae. Any dermatosis is generally less well tolerated in women with premenstrual tension at this time of the cycle. *Acne vulgaris*, rosacea, and cutaneous lupus erythematosus commonly deteriorate. In addition, premenstrual flare-up is recognized in a variety of dermatoses including psoriasis, atopic dermatitis, perioral dermatitis, lichen planus, dermatitis herpetiformis, erythema multiforme, pompholyx, and urticaria [13, 14]. Pemphigoid gestationis occasionally persists in postpartum, often following a pattern of premenstrual exacerbations. Herpes simplex and aphthosis, although frequently recurrent, are not strictly governed by the ovarian cycle.

### 3.5. Autoimmune Progesterone Dermatitis

AIPD is a term covering a variety of skin diseases characterized by cyclic recurrent premenstrual exacerbations related to fluctuations in serum progesterone levels. Progesterone autoantibody production as a response to either administered or endogenous progesterone is involved in AIPD pathogenesis [15–17]. This condition has been exclusively reported only in ovulating women. Two thirds of cases have been exposed to progesterone ingestion under oral contraception prior to the eruption. The mechanism by which women become sensitive to progesterone remains uncertain. A hypothesis involves previous intake of progestogens that induced sensitization to endogenous progesterone. It is suggested that some synthetic progesterone derivatives are sufficiently antigenic to act as a stimulus for antibodies cross-reacting with natural progesterone and perpetuate the immune premenstrual response. However, not all women with AIPD had been previously exposed to synthetic progestogens. The evidence for autoimmunity to progesterone is supported by (a) positive controlled intradermal tests and response to anovulatory drugs, (b) recurrence on intramuscular or oral progesterone challenge, and (c) demonstration of circulating antibodies to progesterone [18].

The nature of the immune reaction occurring to progesterone in AIPD remains unclear. Intradermal progesterone tests showed in some cases an immediate urticarial reaction. However, more frequently a delayed hypersensitivity reaction was mentioned. Type III immune-complex-mediated hypersensitivity was likely involved in some patients developing circulating IgG antibodies. Reactions were often difficult to interpret with confidence. False positive reactions possibly occur, and skin necrosis at test sites is considered as an adverse event. Progesterone challenge during the first half of the ovarian cycle is probably a reliable induction procedure. Any progesterone challenge producing a flare of the eruption represents a substantial evidence for progesterone sensitivity.

 AIPD is a rare condition exhibiting diverse clinical presentations (Table 3). Those most frequently encountered are unspecified dermatitis, erythema multiforme, urticaria, pompholyx, stomatitis, and a dermatitis herpetiformis-like eruption [19]. Clinical and histopathological features do not identify and distinguish the AIPD-related conditions. The onset of AIPD is usually in early adult life, occasionally after a regular pregnancy. The duration of the disorder is variable, with frequent spontaneous remissions. The dermatitis typically flares during the second half of the ovarian cycle, with a premenstrual peak and rapid resolution within a few days of menstruation. Skin lesions are less florid, or skin is cleared during the first half of the cycle. Any AIPD shows cyclic premenstrual exacerbations of the eruption corresponding to the postovulation rise in serum progesterone. 

Rare cases of AIPD have appeared or worsened during pregnancy, with or without postpartum premenstrual cyclic flare up. This condition is tentatively explained by the steady rise in the levels of progesterone and estrogen throughout pregnancy. Other rare cases were associated with spontaneous abortion. However, spontaneous improvement or clearing during pregnancy is reported in other cases.

 Many immune reactions commonly improve during pregnancy, in relation with the reduced maternal immune status during pregnancy and the elevated cortisol levels. It is suggested that the gradual rise in the progesterone levels during pregnancy brings about hormonal desensitization in some individuals.

 The majority of AIPD cases fail to respond to conventional skin treatment modalities, although oral prednisolone in moderately high doses commonly brings about control. Many cases respond well to conjugated estrogens, presumably by suppression of ovulation, thus preventing the postovulatory rise in progesterone. However, in practice estrogen therapy is often not appropriate regarding the age of the usual patients. When estrogen therapy is unsuccessful, the antiestrogen/anovulatory drug tamoxifen is indicated. 

 A pharmacological oophorectomy relies on subcutaneous injections of an antagonist of luteinizing hormone-releasing hormone (LHRH) over a six-month period. Goserelin in a dosage of 3.6 mg by subcutaneous injection may be used for this. When the patient is severely affected, surgical oophorectomy has been occasionally recommended for controlling AIPD [20].

## 4. Conclusion

In some women, endocrine fluctuations during the ovarian cycle possibly exert prominent detrimental effects on the skin. Catamenial acne and AIPD are typical cyclic skin disorders recognized to alter the quality of life. They represent gender-directed facets of chronobiology potentially starting in early adulthood and waning at menopause.

As a consequence of the diversity of endocrine signals to the sebaceous apparatus, sebum excretion varies according to age, gender, pregnancy, and postmenopause. However, at any given age, the sebum excretion rate differs between individuals over a wide range, both in men and women. In addition, a huge overlap exists between data gained in both genders. Hence, it is not the amount of circulating androgens but rather the receptivity of the target tissues that accounts for interindividual differences in sebum excretion. Additional factors are likely operative as well.

## Figures and Tables

**Table 1:** Premenstrual syndrome.

Breast fullness/tenderness
Constipation, frequency of micturition
Edema, weight gain
Excitability, irritability
Headache, migraine
Lethargy, malaise, depression
Nausea, vomiting
Seborrhea, acne

**Table 2:** Chronic dermatoses possibly exhibiting premenstrual flare-up.

Acne
Atopic dermatitis
Dermatitis herpetiformis
Erythema multiforme
Lichen planus
Lupus erythematosus
Pemphigoid gestationis
Pompholyx
Pruritus vulvae
Psoriasis
Rosacea
Urticaria

**Table 3:** Dermatoses possibly corresponding to an autoimmune progesterone dermatitis.

Dermatitis herpetiformis
Erythema multiforme
Nonspecific papular erythema
Pompholyx
Stomatitis
Urticaria
